# Heart filling exceeds emptying during late ventricular systole in patients with systolic heart failure and healthy subjects – a cardiac MRI study

**DOI:** 10.1111/cpf.12555

**Published:** 2018-12-02

**Authors:** Marcus Carlsson, Martin Ugander, Mikael Kanski, Rasmus Borgquist, Ulf Ekelund, Håkan Arheden

**Affiliations:** ^1^ Department of Clinical Sciences, Clinical Physiology Skane University Hospital Lund University Lund Sweden; ^2^ Department of Clinical Sciences, Cardiology Skane University Hospital Lund University Lund Sweden; ^3^ Department of Clinical Sciences, Emergency Medicine Skane University Hospital Lund University Lund Sweden

**Keywords:** cardiac output, cardiac pumping, diastolic dysfunction

## Abstract

**Background:**

Total heart volume (THV) within the pericardium is not constant throughout the cardiac cycle and THV would intuitively be lowest at end systole. We have, however, observed a phase shift between ventricular outflow and atrial inflow which causes the minimum THV to occur before end systole. The aims were to explain the mechanism of the late‐systolic net inflow to the heart and determine whether this net inflow is affected by increased cardiac output or systolic heart failure.

**Methods and Results:**

Healthy controls (*n* = 21) and patients with EF<35% (*n* = 14) underwent magnetic resonance imaging with flow measurements in vessels to and from the heart, and this was repeated in nine controls during 140 μgram kg^−1^ min^−1^ adenosine infusion. Minimum THV occurred 78 ± 6 ms before end of systolic ejection (8 ± 1% of the cardiac cycle) in controls. The late‐systolic net inflow was 12·3 ± 1·1 ml or 6·0 ± 0·5% of total stroke volume (TSV). Cardiac output increased 66 ± 8% during adenosine but late‐systolic net inflow to the heart did not change (*P* = 0·73). In patients with heart failure, late‐systolic net inflow of the heart′s left side was lower (3·4 ± 0·5%) compared to healthy subjects (5·3 ± 0·6%, *P* = 0·03).

**Conclusions:**

Heart size increases before end systole due to a late‐systolic net inflow which is unaffected by increased cardiac output. This may be explained by inertia of blood that flows into the atria generated by ventricular systole. The lower late‐systolic net inflow in patients with systolic heart failure may be a measure of decreased ventricular filling due to decreased systolic function, thus linking systolic to diastolic dysfunction.

## Introduction

The total heart volume (THV) has been shown to decrease during systole by 4%–11% in man (Bowman & Kovacs, 2003; Carlsson *et al*., 2004, 2005; Steding‐Ehrenborg *et al*., 2013a,b). There is a decrease in the content of the pericardial sac during systole, the so‐called total heart volume variation (THVV), caused by the difference in flow between blood ejected into the great arteries and the blood drawn into the atria from the caval and pulmonary veins by the atrioventricular plane displacement (AVPD) (Lundback, 1986; Carlsson *et al*., 2007a,b; Steding‐Ehrenborg *et al*., 2013a,b) (Waters *et al*., 2005). In other words, the ejecting ventricles manage to eject a greater volume than what the descent of the closed AV‐plane pulls into the atria. This is performed by inward displacement of the epicardial surface of the ventricles (Waters *et al*., 2005; Carlsson *et al*., 2007a,a,b,b). This is also evident from the biphasic inflow to the atria in both diastole and systole but a monophasic outflow from the ventricles only during systole. Thus, the atrial reservoir function (Nishikawa *et al*., 1994; Prioli *et al*., 1998; Tseng *et al*., 2002; Bowman & Kovacs, 2004; Gaynor *et al*., 2005) is coupled to the longitudinal ventricular function through the AVPD. However, the role of AVPD in cardiac pumping has been debated (Arutunyan, 2015; Arvidsson *et al*., 2015) (Carlsson *et al*., 2018) (Stokke *et al*., 2017). The time point of the lowest THV has been described to coincide with the end of systole (Carlsson *et al*., 2004), but close inspection of the flow measurements published show that the inflow to the heart exceeds the outflow prior to the end of systolic ejection. This results in an increase in THV before the end of systolic ejection, which means that the lowest THV does not coincide with end systole. The increase in THV between minimum THV and end systole is caused by this late‐systolic net inflow which must occur in the atria because of ongoing ventricular ejection and closed atrioventricular (AV) valves. To our knowledge, the presence of a late‐systolic net inflow has not previously been described or quantified. The inflows and outflows of the heart are of great importance as the generation of pressurized flow is the main function of the heart. Thus, the rationale of this study was to provide mechanistic insights into basic cardiac pumping physiology and the pathophysiology in heart failure patients through the quantification of the phase shift between inflow and outflow of the heart. We hypothesized that this phase shift would be greater in patients compared to controls and that an increase of cardiac output in controls would decrease this phase difference and thus increase the synchronizing of the inflow and outflow to the heart. Adenosine increases cardiac output to counter the decreased peripheral resistance to maintain systemic blood pressure. Adenosine thus offers the possibility to study the effect of increased cardiac flow through changes in afterload triggering the sympathetic nervous system.

Therefore, the aims of this study were to describe and quantify the late‐systolic net inflow causing an increase in heart volume before the end of systolic ejection, and to determine whether this is affected by increased cardiac output in healthy subjects or in patients with left ventricular systolic heart failure.

## Materials and methods

### Study population and design

The study was approved by the regional ethics review board in Lund. Twenty‐one healthy volunteers (nine females) with a blood pressure <140/90, normal resting ECG and no cardiovascular medication were included in the study, and written informed consent was obtained. All subjects underwent cardiac magnetic resonance imaging in the supine position with flow and cine imaging. Eight subjects additionally underwent flow and cine imaging during 140 microgram kg^−1^ min^−1^ adenosine infusion to increase cardiac output with vasodilatation. The adenosine examinations were performed with 24‐h caffeine abstinence as previously described (Carlsson *et al*., 2015).

Patients (*n* = 14, five females) with decreased systolic function on echocardiography (EF <35%) investigated for possible treatment of dyssynchrony were included. All patients had left bundle branch block, ventricular dyssynchrony and none had significant valvular disease. Ten patients had ischaemic and four patients non‐ischaemic dilated cardiomyoatphy. All patients had betablocker and either angiotensin converting enzyme inhibitor or angiotensin II receptor type 1 blockade.

### Magnetic Resonance Imaging

A 1.5 T MRI‐scanner with a cardiac synergy coil was used (Philips Intera CV, Philips, Best, the Netherlands). All images were evaluated using freely available software (Segment 1·6‐2·0 http://segment.heiberg.se) (Heiberg *et al*., 2010).

### Cine imaging

Image acquisition and analysis was performed as previously described (Carlsson *et al*., 2007a,b). In short, steady‐state free‐precession cine images with retrospective ECG triggering were acquired. The THV was measured by outlining the pericardial borders of the total heart in short‐axis images, and AVPD was measured in the long‐axis images (Carlsson *et al*., 2004, 2007a,a,b,b). The left ventricular (LV) and right ventricular (RV) stroke volume (SV) was calculated by delineation of the endocardial borders of the LV and RV in short‐axis cine images (Pennell, 2002; Shors *et al*., 2004). The total stroke volume (TSV) was calculated by adding the LVSV and RVSV, obtained from volumetric measurements of short‐axis images. The rate of systolic AVPD was calculated as the systolic AVPD divided by time of systolic AVPD.

### Flow imaging

Flow images of all vessels leading to (caval and pulmonary veins) and from (aorta and the pulmonary trunk) the heart were acquired and analysed as previously described (Carlsson *et al*., 2007a,b). In short, a free breathing fast field echo velocity encoded sequence with retrospective ECG triggering was used. In healthy subjects undergoing adenosine a breath‐hold segmented turbo field, echo velocity encoded sequence with retrospective ECG triggering was used to decrease scan time during adenosine infusion. All inflows (ml s^−1^) into the heart (caval and pulmonary veins) and outflows from the heart (aorta and pulmonary trunk) were added to determine total inflow and outflow over the cardiac cycle. In patients, only flows of the left side of the heart were collected to decrease MR scanning time.

### Late‐systolic net inflow

**Figure 1:**
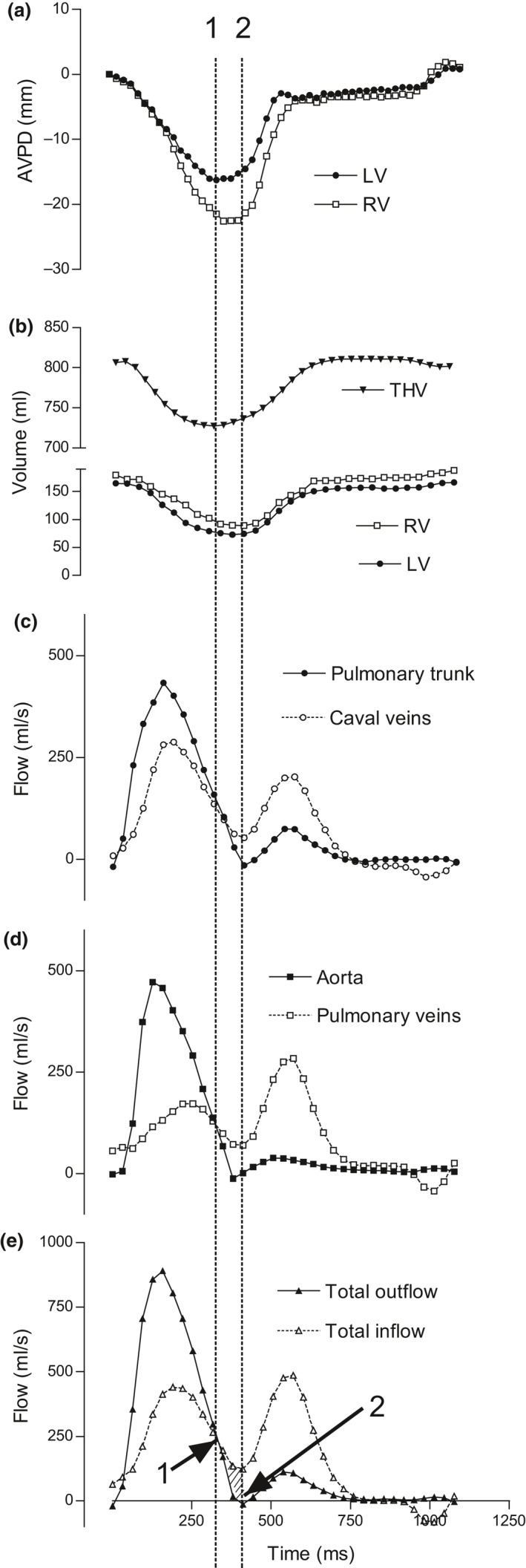
Data from one typical subject over the cardiac cycle. (a). Atrioventricular plane displacement (AVPD) of the left ventricle (LV) and the right ventricle (RV). (b). Total heart volume (THV) and volume of the LV and RV. Note that the THV in the latter part of diastole is greater that the THV in end diastole. (c). Flow in the pulmonary trunk and the combined flow of the inferior and superior caval veins. (d). Flow in the aorta and the combined flow of the pulmonary veins. There is negative flow at the end of diastole in all veins, coinciding with atrial contraction as seen in (a), and resulting in the decrease of the THV in late diastole as seen in (b). (e). The sum of the outflow (aorta and pulmonary trunk) from and inflow (caval and pulmonary veins) to the heart. The dashed vertical line 1 indicates the time of the lowest THV as seen in B. This also corresponds to the time when total inflow is equal to total outflow as seen in E (arrow 1). The dashed vertical line 2 indicates the end of systolic ejection defined as the end of systolic forward flow in the outflow (aorta and pulmonary trunk) from the heart. This is seen where the solid line in E crosses the *x*‐axis (arrow 2). The inflow to the heart is greater than the outflow from the heart during the time interval between lines 1 and 2, resulting in a late‐systolic net inflow (marked by diagonal lines in panel E). This results in an increase in THV which occurs prior to the completion of systolic ejection. Note that the lowest THV does not correspond to the lowest LV and RV volume, nor to the lowest point of AVPD. This means that the THV starts to increase before the end of systole.

The late‐systolic net inflow was defined as the increase in THV which occurred between minimum THV and the end of systolic ejection as calculated from the flow data. The total inflow and outflow was plotted for each subject, and the time point when the outflow was equal to the inflow in late systole was identified (line 1 and arrow 1 in Fig. 1). This time point in the cardiac cycle corresponds to the lowest THV (Fig. 1b). Next, the time point when the combined systolic flow of the aorta and pulmonary trunk reached 0 ml s^−1^ was identified (line 2 and arrow 2 in Fig. 1). This time point in the cardiac cycle corresponds to the end of systolic ejection, or the end of ventricular systole. The difference between inflow and outflow between these time points was used to calculate the volume increase in THV during late systole or late‐systolic net inflow. The magnitude of the late‐systolic net inflow was expressed in absolute volume as well as per cent of both THV, THVV and stroke volume. Furthermore, late‐systolic net inflow was quantified for the left and right side of the heart using pulmonary veins/aorta and caval veins/pulmonary artery, respectively.

### Statistical analysis

Continous variables are expressed as mean ± standard error of the mean (SEM). Non‐parametric tests were used to test the significance of the differences between variables as normal variation could not be assumed. The Wilcoxon test was used for comparison of inter‐subject differences and Mann–Whitney test for comparsion between subjects. Results with a *P*‐value <0·05 were defined as statistically significant. The relationship between variables was determined by linear regression analysis.

## Results

### Study population

**Figure 2:**
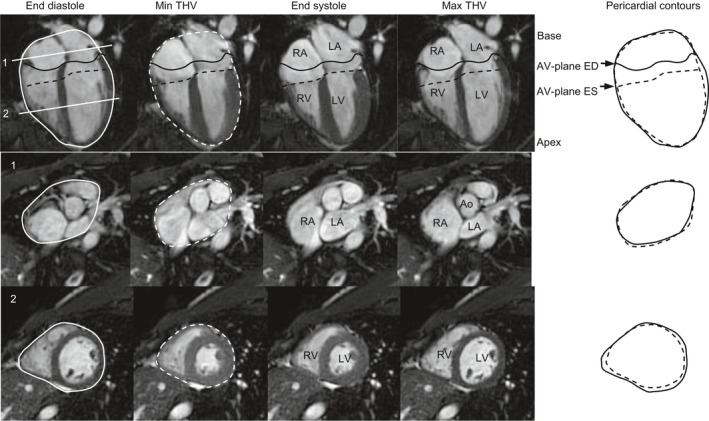
Inner and outer volume changes throughout the cardiac cycle. MR images of the subject in Fig 1 are shown in the following phases of the cardiac cycle: End diastole (ED), with closure of atrioventricular (AV) valves; Minimum total heart volume (Min THV), the lowest total heart volume; End systole (ES): conclusion of systolic ejection out of the heart through the aorta and pulmonary trunk; Maximum total heart volume (Max THV), the largest total heart volume corresponding to a time point just before atrial contraction. The figure shows the left ventricle (LV), right ventricle (RV), left atrium (LA), right atrium (RA) and aorta (Ao). The white lines 1 and 2 in the top left four‐chamber image indicate the positions of the short‐axis images in the numbered rows 1‐2. The black solid lines indicate the position of the atrioventricular (AV) valves in end diastole, and the black broken lines indicate the position of the AV valves in end systole. The solid circular contours denote the pericardial contour of the heart at ED. The broken circular contours denote the pericardial contour of the heart at the lowest total heart volume (THV). Note that the AV‐plane has not reached its most apical position at the time of minimum THV. End systole with the farthest excursion of the AV‐plane occurs ofter minimum THV.

The physiological characteristics of the volunteers and patients are listed in Table 1. Total heart volume in healthy subjects was 956 ± 49 ml and THVV 7·2 ± 0·4%. Figure 1 shows an example of the results from the in and outflows, to and from the heart in one subject. Filling rate of the ventricle and diastolic AVPD rate is larger than emptying rate and systolic AVPD rate. The AVPD in late diastole coincides with atrial contraction. Representative MR images in the four‐chamber and short‐axis views from this subject are shown in Fig. 2. Note that the AV‐plane at the minimum THV has not reached its most apical position in the RV. This shows that the minimum THV occurs prior to end systole. Also, note that the AV‐plane at maximum THV has not reached its most basal position (Fig. 1). The ensuing atrial contraction causes the late diastolic AVPD and also results in a decreased THV to the THV at end diastole.

**Table 1:** Results from healthy controls and patients at rest

Parameter	Healthy controls (*n* = 21)	Patients (*n* = 14)	*P*‐value
Age (years)	*32 *±* *2	69 ± 2	<0·001
Heart rate (beats minut^−1^)	62 ± 2	68 ± 3	0·12
Body surface area (m^2^)	1·9 ± 0·0	1·9 ± 0·1	0·65
LV ejection fraction	63 ± 1	28 ± 2	<0·001
LV end‐diastolic volume index (ml m^−2^)	95 ± 3	135 ± 6	<0·001
LV end‐systolic volume index (ml m^−2^)	35 ± 2	99 ± 6	<0·001
Left late‐systolic net inflow (ml)	5·5 ± 0·7	2·2 ± 0·3	<0·001
Left late‐systolic net inflow/stroke volume (%)	5·3 ± 0·6	3·4 ± 0·5	0·03

### Late‐systolic net inflow

The increase in total heart volume during late systole before the end of systolic ejection (late‐systolic net inflow) for both sides of the heart and for the total heart is illustrated in Fig. 1. The late‐systolic net inflow is the difference in inflow and outflow between the time points indicated by the two vertical dotted lines 1 and 2. The late‐systolic net inflow for all healthy subjects was 11·8 ± 1·2 ml (range 3·0–21·6 ml) (Table 1). This corresponds to 5·8 ± 0·5% of the total stroke volume or 18 ± 2% (range 7%–31%) of the THVV. The duration of the late ejection filling was 78 ± 6 ms (range 45–146 ms) or 8 ± 1% of the cardiac cycle (range 5%–15%). There was no correlation between late‐systolic net inflow indexed to stroke volume and heart rate (*P* = 0·88) or duration of systole (*P* = 0·13). Also, the late‐systolic net inflow of the left and right side of the heart did not differ (5·4 ± 0·6 versus 5·4 ± 0·8 ml, *P* = 0·65).

### Adenosine

**Figure 3:**
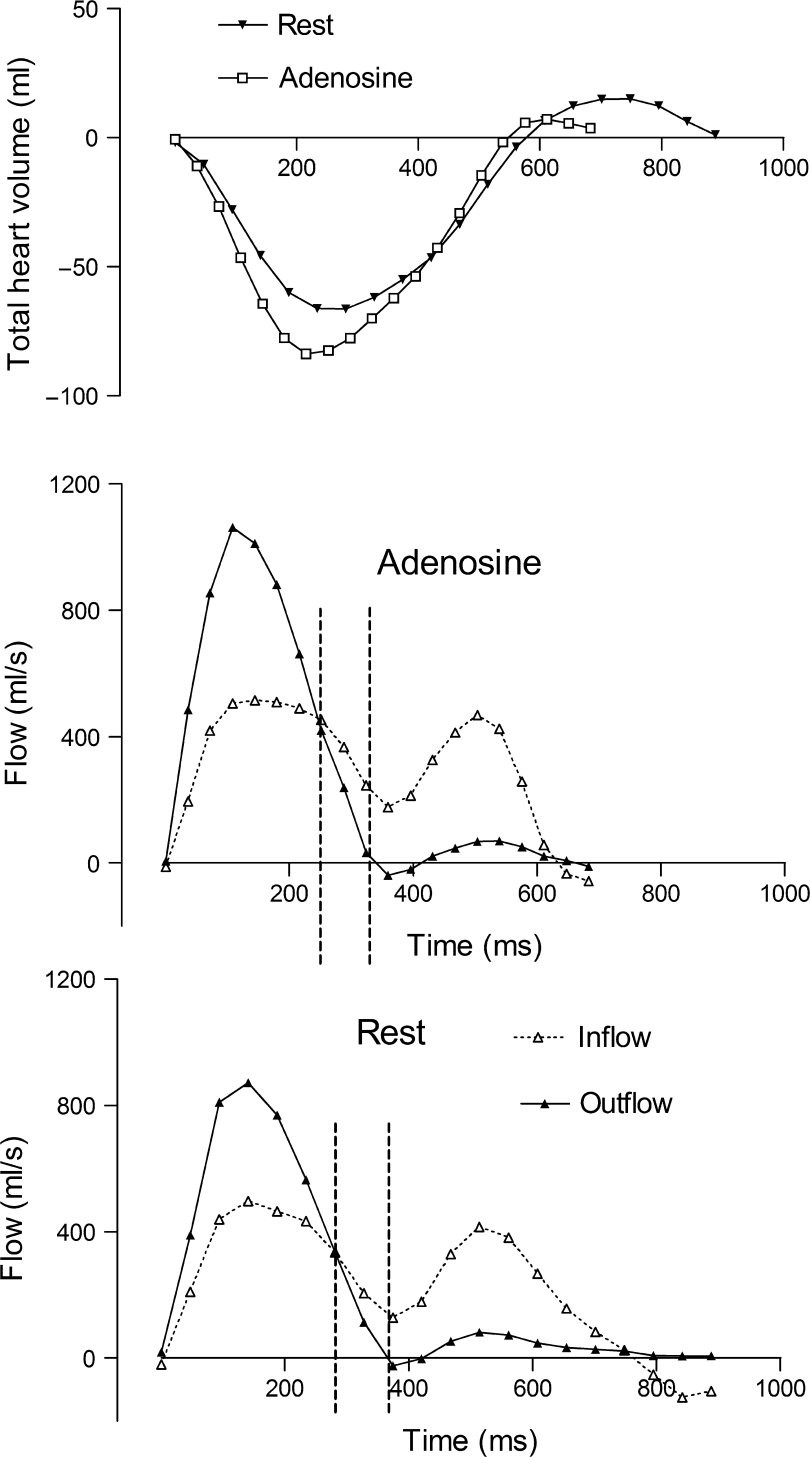
The summed outflows (solid triangles, solid line) and inflows (open triangles, dotted lines) at rest and during adenosine. The top panel shows the resulting total heart volume changes caused by the difference in outflows and inflows. The time for late‐systolic net inflow is indicated in the flow curves with vertical dashed lines. In this patient, there is a small increase in total heart volume variation during adenosine but in average between subjects, this difference was not statistically significant. There were no significant changes in late‐systolic net inflow at rest or during adenosine infusion.

**Figure 4:**
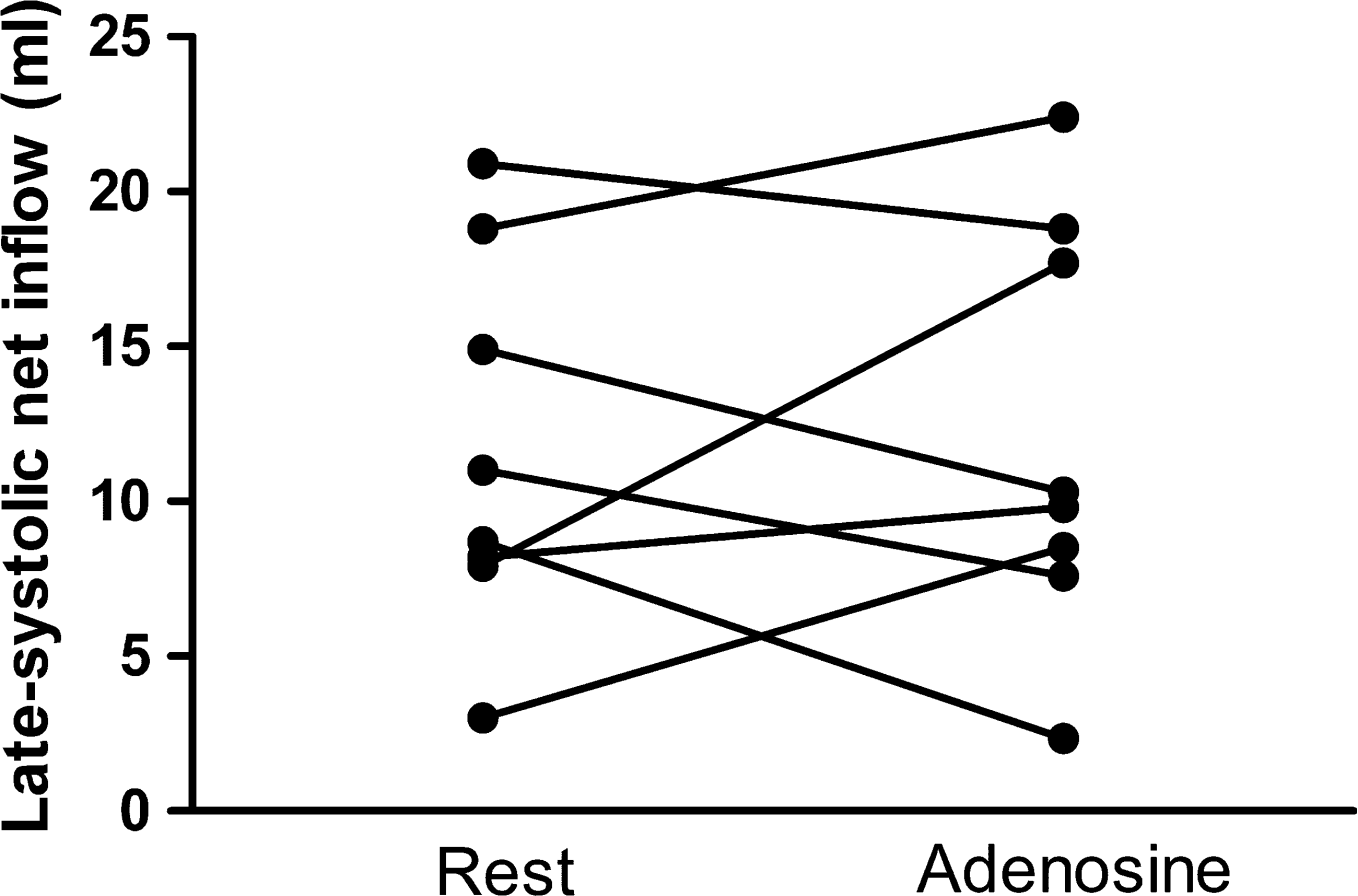
Late‐systolic net inflow in subjects at rest and during adenosine in healthy volunteers.

Adenosine increased cardiac output by 63 ± 9% primarily due to an increase in heart rate of 55 ± 8% and to a lesser extent by an increase in stroke volume of 6 ± 3%. THV, THVV and THVV/TSV did not change during adenosine vasodilatation (Table 2). Figure 3 shows the summed inflow and outflow over the cardiac cycle for one patient at rest and during adenosine, and the time of larger inflow than outflow (time of late‐systolic net inflow) during late systole is indicated by vertical lines similar to Fig. 1. The phase shift of atrial inflow versus ventricular outflow did not change resulting in an unchanged late‐systolic net inflow for the total heart during adenosine (11 ± 2 ml) compared to rest (12 ± 2 ml, *P* = 0·80) (Fig. 4). Separate analyis of the different sides of the heart showed no difference in late‐systolic net inflow during adenosine compared to rest at the left side (5 ± 1 ml versus 6 ± 1 ml at rest, *P* = 0·48) or the right side (5 ± 1 ml versus 5 ± 1 ml at rest, *P* = 0·64).

**Table 2:** Effects of adenosine vasodilatation

Parameter	Rest	Adenosine	*P*‐value
Heart rate (beats minut^−1^)	60 ± 3	91 ± 4	<0·001
Total stroke volume (ml) (aorta and pulmonary)	206 ± 17	219 ± 20	0·04
Total Cardiac output (l min^−1^) (aorta and pulmonary)	12 ± 0	20 ± 2	<0·001
Late‐systolic net inflow (ml)	12 ± 2	12 ± 2	0·80
Late‐systolic net inflow/stroke volume (%)	6 ± 1	6 ± 1	0·90
Total heart volume (ml)	848 ± 75	868 ± 79	0·14
Total heart volume variation (ml)	68 ± 12	72 ± 11	0·47
Total heart volume variation (%)	8 ± 1	8 ± 1	0·71

### Patients with systolic heart failure

**Figure 5:**
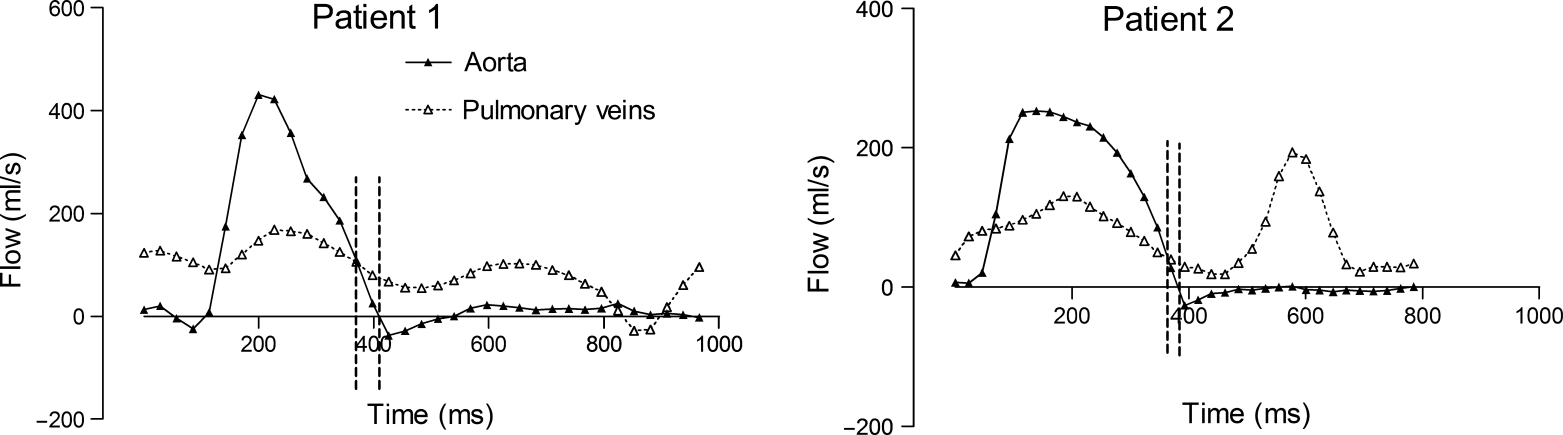
Examples of flows in patients with systolic heart failure. The summed outflows (solid triangles, solid line) and inflows (open triangles, dotted lines) are shown for two patients. The time for late‐systolic net inflow is indicated in the flow curves with vertical dashed lines. Note that despite the difference in flow patterns, there is a late‐systolic net inflow in both patients.

**Figure 6:**
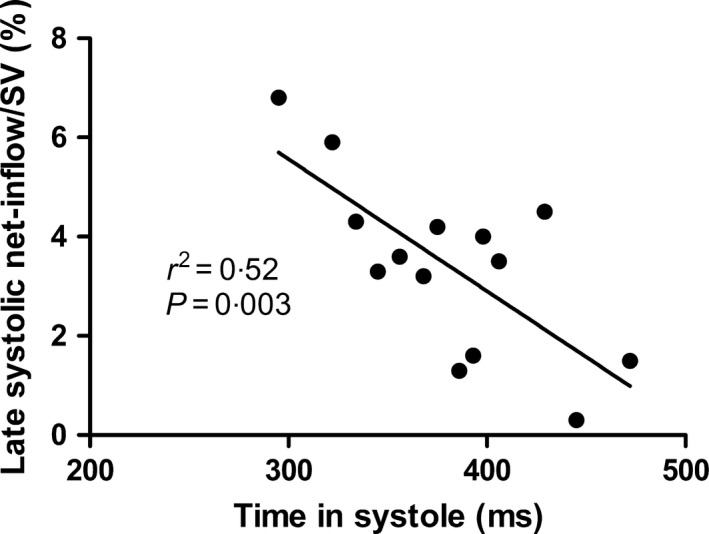
Late‐systolic net inflow normalized to stroke volume (SV) in patients with systolic heart failure showed a strong correlation with the duration of systole.

The in‐ and outflows of the left side of the heart from two patients with systolic heart failure are shown in Fig. 5. Late‐systolic net inflow was lower in patients with systolic heart failure in both absolute volume and indexed to left ventricular stroke volume compared to healthy subjects (Table 1). There was a strong correlation of late‐systolic net inflow normalized to stroke volume and the duration of systole (Fig. 6, *r* ^2^ = 0·52, *P* = 0·003, y = −0·03x + 13·5) in patients, but not in controls (*r* ^2^ = 0·13, *P* = 0·13). There was no correlation in patients between late‐systolic net inflow and ejection fraction (*P* = 0·78), left ventricular volumes (*P* = 0·85) or heart rate (*P* = 0·08).

## Discussion

This study has demonstrated and quantified the presence of a net inflow to the heart during late systole showing that the heart is not smallest at the end of ventricular systole but rather ~80 ms before the end of systolic ejection. This is the result of a phase shift between the rapid systolic ventricular ejection and the resulting atrial filling. We propose that the mechanism for this phase shift is the inertia of the blood in the atria and veins. In patients with systolic heart failure where ejection of blood is slower, the lower inertia does not limit concomitant atrial filling and therefore ventricular ejection and atrial filling is more in phase and the late‐systolic net inflow is lower. This hypothesis was supported by the strong negative correlation between late‐systolic net inflow and systolic duration in patients but the absence of correlation in controls. However, larger studies are needed in patients with heart failure and in particular patients without left bundle branch block. The momentum of blood into the atria towards the atrioventricular valves during end systole might facilitate valve opening and early diastolic filling especially on the right side of the heart where diastolic ventricular suction is less pronounced (Carlsson *et al*., 2012; Arvidsson *et al*., 2013). The lower late‐systolic net inflow in patients with systolic heart failure may serve as a measure of decreased ventricular filling due to decreased systolic function and thus linking systolic to diastolic dysfunction. Finally, this study found that the late‐systolic net inflow was unaffected by altered loading conditions in the form of increased heart rate and cardiac output caused by adenosine vasodilatation.

### Physiologic implications

This study has for the first time demonstrated that the heart begins to increase in size before the end of systolic ejection. The volume difference which was generated between the minimum total heart volume and the end of systolic ejection was quantified to ~20% of the total heart volume variation and is due to inflow of blood into the atria. Notably, the late‐systolic net inflow must be located in the atria because the AV valves are closed during this time period of ongoing ventricular ejection. This late‐systolic atrial filling is caused by flow of blood in the pulmonary and caval veins which is accelerated into the atria due to the suction of ventricular systole (Zhang *et al*., 2008; Steding‐Ehrenborg *et al*., 2013a,b). Furthermore, the systolic longitudinal AVPD must be the primary cause of the late‐systolic net inflow as systolic radial function does not cause atrial filling because of the closed AV‐valves (Carlsson *et al*., 2007a,b). The systolic inflow from the pulmonary veins often have two peaks, where the first (S1) is caused by a drop in left atrial pressure and the second (S2) by the systolic outflow from the right ventricle (Smiseth *et al*., 1998, 1999). The drop in left atrial pressure causing S1 is generated by the AVPD (Steding‐Ehrenborg *et al*., 2013a,b). Diastolic atrial filling is mainly caused by ventricular suction (Zhang *et al*., 2008; Steding‐Ehrenborg *et al*., 2013a,b); however, the kinetic energy from inflowing venous blood generated during systole may also affect diastolic filling (Carlsson *et al*., 2012; Arvidsson *et al*., 2013) (Steding‐Ehrenborg *et al*., 2016). The coupling between systolic AVPD and late‐systolic net inflow may be that kinetic energy from the inflowing venous blood is generated during early systole, and the inertia of the blood from the veins causes continued filling in late systole and ultimately early diastole (Noble, 1968). This possible coupling of systolic ejection and diastolic filling also may suggest that diastolic dysfunction may be a mild systolic dysfunction manifested during diastole. The reduction in systolic function in patients would thereby result in a decrease in kinetic energy of the inflowing venous blood, explaining the reduced late‐systolic net inflow in the present study and may be coupled to the reduced diastolic ventricular filling rate. This hypothesis is supported by previous studies showing a coupling between systolic and early diastolic function (Kilner *et al*., 2000; Winter *et al*., 2004) and decreased longitudinal systolic function in patients with diastolic dysfunction (Nikitin *et al*., 2002; Yip G*, et al*. 2002; Yip G. W.*, et al*. 2002). Thus, the late‐systolic net inflow may be a novel sensitive measure of diastolic dysfunction. Left ventricular kinetic energy during diastole was recently shown to have deranged patterns in heart failure patients (Kanski *et al*., 2015). Another, possible explanation for decreased late‐systolic net inflow in patients may be that a preserved right ventricular function results in the forward‐going propagation wave contributing to atrial filling (Smiseth *et al*., 1999) in mid‐systole as the decreased left ventricular function leads to a longer ejection phase.

The pumping heart is adapted for high flows during exercise. During exercise with increased flows, the heart functions more like a turbine with continuous flow and at rest it functions more like a displacement pump with acceleration and deceleration of blood into and out of the heart as well as between the atria and ventricles. Therefore, a more matched inflow and outflow during increased flow rates compared to rest, when there is a phase shift between inflow and outflow, could be expected. However, in the present study, there was no difference in phase shift between inflow and outflow using adenosine vasodilatation to pharmacologically achieve increased flow and thus no difference in outer heart volume variations. Interestingly, previous findings during exercise in fact showed increased outer heart volume variations, meaning larger phase shifts between inflows and outflows are present during exercise compared to rest (Steding‐Ehrenborg *et al*., 2013a,b).

Ballistocardiography has been used to detect the displacement of the body from the ejection of blood and contraction of the heart and thus detects inertial cardiohemic oscillations (Giovangrandi *et al*., 2011; Vogt *et al*., 2012). The late‐systolic net inflow caused by inertia may therefore be a part of the oscillations detected by ballistocardiography, specifically the K‐wave. Of note, the physiological cause of the oscillations has not been fully clarified (Giovangrandi *et al*., 2011).

### Further studies

This is a first description of the late‐systolic net inflow, and further studies are needed to establish if the late‐systolic net inflow is helpful as a measure of diastolic function and dysfunction and if there is a prognostic significance of alterations of this meausure. If the late‐systolic net inflow is decreased, this may indicate a systolic cause of diastolic dysfunction. Studies in patients with valvular heart disease can be performed to show whether the late‐systolic net inflow affects valvular function.

### Limitations

Cardiac catheterization was not performed, and therefore, pressure measurement are not available. Flow measurements of the right side of the heart were not collected in patients to minimize MR scan time. Patients with heart failure had left bundle branch block and dyssynchrony. Further studies need to investigate whether the results with a decreased late‐systolic net infow can be extended to patients with heart failure but no branch block.

## Conclusions

The smallest heart volume is prior to end systole as a result of a late‐systolic net inflow due to a phase shift between ventricular outflow and atrial inflow. This may be explained by the blood inertia as this physical property of blood result in a slower atrial filling by longitudinal shortening of the ventricles than ventricular ejection of blood. The late‐systolic net inflow was unaffected by increased cardiac output but lower in patients with systolic heart failure. This may be caused by inertia of blood that flows into the atria generated by ventricular systole. Patients with systolic heart failure have lower late‐systolic net inflow, and this may be explained by a more sluggish ventricular ejection of blood and a larger role of increased filling pressures for atrial filling compared to healthy subjects. These mechanisms may have relevance for diastolic dysfunction, linking systolic and diastolic function as well as valvular heart disease as the momentum of the late‐systolic net inflow towards the valve may facilitate opening.

## Funding

This study has been funded in parts by grants from the the Swedish Research Council, Swedish Heart Lung Foundation, the Swedish Medical Association, the Faculty of Medicine, Lund University, Sweden and the Region of Skåne, Sweden.

## Conflicts of interest

Nothing to declare.
